# Development of the rodent prefrontal cortex: circuit formation, plasticity, and impacts of early life stress

**DOI:** 10.3389/fncir.2025.1568610

**Published:** 2025-03-26

**Authors:** Xinyi Chen, Yuri Kim, Daichi Kawaguchi

**Affiliations:** Graduate School of Pharmaceutical Sciences, The University of Tokyo, Tokyo, Japan

**Keywords:** prefrontal cortex, critical period, plasticity, neural circuit, development, epigenetics, early life stress, neurodevelopmental disorder

## Abstract

The prefrontal cortex (PFC), located at the anterior region of the cerebral cortex, is a multimodal association cortex essential for higher-order brain functions, including decision-making, attentional control, memory processing, and regulation of social behavior. Structural, circuit-level, and functional abnormalities in the PFC are often associated with neurodevelopmental disorders. Here, we review recent findings on the postnatal development of the PFC, with a particular emphasis on rodent studies, to elucidate how its structural and circuit properties are established during critical developmental windows and how these processes influence adult behaviors. Recent evidence also highlights the lasting effects of early life stress on the PFC structure, connectivity, and function. We explore potential mechanisms underlying these stress-induced alterations, with a focus on epigenetic regulation and its implications for PFC maturation and neurodevelopmental disorders. By integrating these insights, this review provides an overview of the developmental processes shaping the PFC and their implications for brain health and disease.

## Introduction

**Figure 1:**
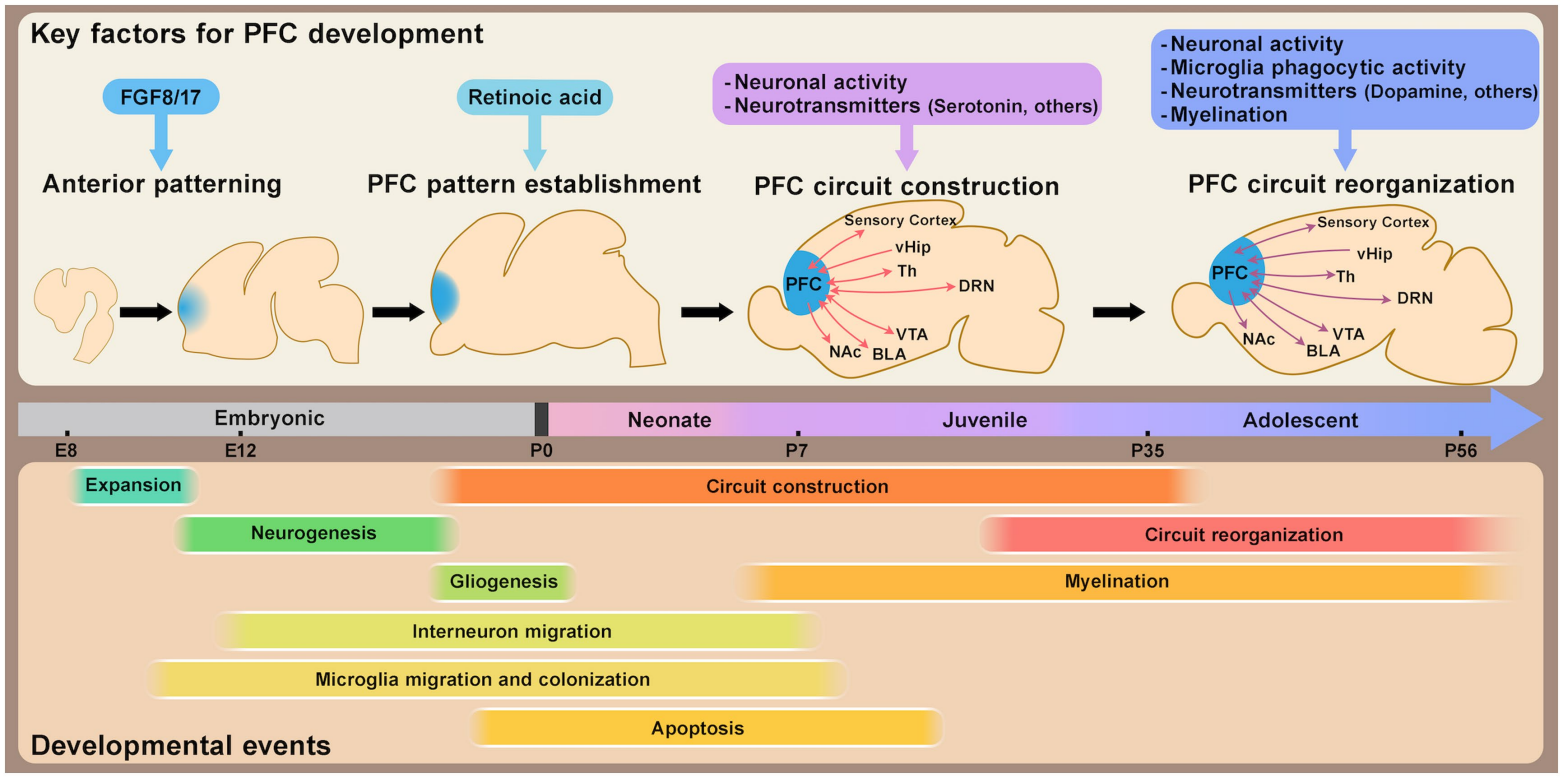
Schematic timeline of mouse prefrontal cortex (PFC) development. This schematic illustrates the approximate timeline of key developmental events shaping PFC structural and circuit organization, along with major factors regulating PFC development and maturation. PFC patterning is largely established by the perinatal stage. Circuit formation progresses from the neonatal stage through adolescence, with synaptic refinement and circuit reorganization occurring during both the juvenile and adolescent stages. Abbreviations: vHip, ventral hippocampus; Th, thalamus; DRN, dorsal raphe nucleus; VTA, ventral tegmental area; BLA, basolateral amygdala; NAc, nucleus accumbens; E, embryonic day; P, postnatal day.

The prefrontal cortex (PFC) is a brain region located in the anterior part of the frontal lobe. In primates, including humans, the PFC is subdivided into distinct subregions such as the medial PFC (mPFC), lateral PFC (lPFC), and orbitofrontal cortex (oFC), each of which contributes to higher-order brain functions, including decision-making, social behavior, memory processing, attentional regulation, and emotional control (Siddiqui et al., 2008; Kolk and Rakic, 2022; Preuss and Wise, 2022). In rodents, the PFC is thought to be composed of the mPFC [infralimbic cortex (IL), prelimbic cortex (PL), and anterior cingulate cortex (ACC)] and oFC, but probably lacking the anatomical equivalent of dorsolateral PFC in primates (Le Merre et al., 2021; Kolk and Rakic, 2022). The PFC integrates and processes information from a wide range of brain regions (Figure 1), enabling it to coordinate functions essential for adaptive behavior (Miller and Cohen, 2001; Friedman and Robbins, 2022). Dysfunction of the PFC has been implicated in the pathophysiology of various neuropsychiatric disorders, including schizophrenia, depression, and autism spectrum disorder (Siddiqui et al., 2008; Xu et al., 2019).

Despite significant evolutionary diversity, certain subdivisions of the PFC demonstrate structural homology across mammals, including humans, non-human primates, and rodents (Preuss and Wise, 2022). In humans, the PFC is particularly enlarged, with its proportion relative to total brain volume being notably greater than in other mammals, even among primates (Donahue et al., 2018). This unique expansion is thought to support advanced cognitive abilities. Although the rodent PFC differs substantially from that of primates, functional similarities in certain circuits, such as the prefrontal-hippocampal circuit for working memory and the prefrontal-amygdala circuit for social behaviors (Tsutsui et al., 2016; Gangopadhyay et al., 2021), provide valuable insights into its conserved functions across species (Ongür and Price, 2000; Euston et al., 2012; Carlén, 2017; Chini and Hanganu-Opatz, 2021). Additionally, the feasibility of manipulation with advanced genetic and molecular tools makes rodents, particularly mice, an indispensable model for studying PFC circuits and functions. Recent studies also demonstrate that rodent models effectively replicate neural circuits and behaviors relevant to neurodevelopmental disorders, underscoring their importance in PFC research (Schubert et al., 2015; Chini and Hanganu-Opatz, 2021). This review draws primarily on findings from rodent studies, specifically in mice and rats, to outline how the structure and circuits of the PFC are established during development.

Early life stress (ELS), such as abuse or social isolation during infancy, childhood, or adolescence, has been linked to an increased risk of psychiatric disorders, including depression, anxiety, impaired social skills, and memory deficits later in life. Studies in both humans and rodents suggest that specific developmental windows are particularly vulnerable to the effects of stress (Schroeder et al., 2018; Nestler and Russo, 2024). The PFC, characterized by its protracted maturation timeline and heightened postnatal plasticity (Chini and Hanganu-Opatz, 2021; Klune et al., 2021), is believed to be especially susceptible to stress during these critical periods. This vulnerability underscores the potential impact of ELS on mental health. This review provides an overview of PFC circuit maturation mechanisms across distinct postnatal stages and explores how ELS disrupts PFC structure, circuitry, and function. The long-term effects of stress may involve epigenetic modifications, which have the potential to induce persistent changes in gene expression. Notably, findings from genome-wide association studies (GWAS) and related analyses indicate that genetic risk factors for psychiatric disorders—such as autism spectrum disorders, schizophrenia, and intellectual disabilities—are enriched in genes associated with epigenetic regulation and transcriptional control (Najmabadi et al., 2011; Satterstrom et al., 2020; Singh et al., 2022), highlighting their potential role in PFC development and disease vulnerability. Building on these findings, this review also examines the interplay between epigenetic regulation, PFC maturation, and stress responses.

### Patterning, cell type specification, and cytoarchitecture of the prefrontal cortex

The patterning of the cerebral cortex, which is largely conserved across mammalian species (Krubitzer and Seelke, 2012), is established primarily by morphogen concentration gradients that develop along the anterior–posterior and medial-lateral axes during early embryonic development (O’Leary et al., 2007; Greig et al., 2013; Cadwell et al., 2019). At this stage, neural progenitor cells are in an expansion phase, undergoing symmetric cell division to increase their population before differentiating into specialized cell types (Miyata et al., 2010; Greig et al., 2013). The expression patterns of transcription factors in neural progenitor cells, shaped by these morphogen gradients, lay the foundation for cortical patterning. In the anterior region of the mouse brain, including the prefrontal cortex (PFC), fibroblast growth factors (FGFs), such as FGF8 and FGF17, play critical roles. FGF8 is primarily associated with anteriorization (Fukuchi-Shimogori and Grove, 2001), while FGF17 is involved in the compartmentalization of anterior subdivisions (Cholfin and Rubenstein, 2007) (Figure 1). Recent studies have highlighted the role of retinoic acid (RA) signaling in establishing PFC patterning from the embryonic stage to the perinatal period, and subsequently affecting synaptogenesis and thalamo-prefrontal connectivity (Shibata et al., 2021a). Interestingly, RA signaling appears to be differentially regulated in mice and humans, with evolutionary changes in the enhancer region of *Cbln2*, a PFC marker, suggested to contribute to interspecies differences in PFC patterning and layer organization (Shibata et al., 2021b) (Figure 1). Since variations in PFC size and organization are often observed in patients with neurodevelopmental disorders (Glahn et al., 2008; De La Fuente et al., 2013; Bakhshi and Chance, 2015; Courchesne et al., 2019; Hashem et al., 2020; Zhao et al., 2022), understanding the mechanisms underlying PFC patterning could provide insights into the pathophysiology of these conditions.

The specification of cell types in the PFC follows a developmental program similar to other cortical areas. After the expansion phase, neural stem cells undergo the neurogenic phase, producing neurons mainly through asymmetric division, followed by the gliogenic phase, where glial cells such as astrocytes or oligodendrocytes are generated (Miyata et al., 2010; Greig et al., 2013). The production of deeper cortical layers precedes that of upper layers, reflecting a well-defined temporal sequence (Greig et al., 2013). Excitatory neurons in each layer of the PFC eventually form layer-specific connections to different brain regions governing specific functions (Murugan et al., 2017). From an evolutionary perspective, significant differences in the granular layer 4 (L4) are evident among species (Le Merre et al., 2021; Preuss and Wise, 2022). In primates, L4 in the PFC is characterized by a well-defined granular cell layer as in other cortical areas, but the granular L4 is largely absent in the PFC of rodents. In addition to excitatory neurons, cortical interneurons, which originate from the ventral telencephalon, migrate into the cortical layers during the perinatal stage (Wamsley and Fishell, 2017; De Marco García and Fishell, 2024), affecting excitatory-inhibitory balance later in postnatal stages (Caballero et al., 2021). Microglia, known for their roles in synaptic development and plasticity (Wu et al., 2015), invade the cortical parenchyma early during embryonic development and continue to proliferate and organize postnatally in mice (Thion et al., 2018). By approximately one or two weeks after birth in rodents, the fundamental cell types in the cerebral cortex are established and their migration is completed.

The cell types mentioned above (e.g., excitatory neurons, inhibitory neurons, and astrocytes) are known to be further classified into subtypes based on factors such as gene expression patterns and their localization within specific cortical layers (Greig et al., 2013; Lanjakornsiripan et al., 2018; De Marco García and Fishell, 2024). While differences in subtypes or gene expression signatures across cortical areas were previously not well understood, recent technological advancements, such as single-cell and spatial transcriptomics, have begun to provide new insights into how diverse cell types acquire region-specific characteristics in humans, macaques, and mice (Nowakowski et al., 2017; Tasic et al., 2018; Bhaduri et al., 2021; Chen et al., 2024; Qian et al., 2024). Although the mechanisms driving area-specific cell type diversification and maturation during postnatal development remain incompletely understood, current evidence suggests that area-specific traits, including those of the PFC, motor, somatosensory, parietal, temporal, and primary visual cortices, first emerge in neural progenitors and become progressively more distinct during differentiation and maturation in the human fetal brain (Nowakowski et al., 2017; Bhaduri et al., 2021). Ongoing single-cell transcriptomic studies that span distinct stages of postnatal development into adulthood (Zhong et al., 2018; Bhattacherjee et al., 2019, 2023; Ortiz et al., 2020; Joglekar et al., 2021) offer valuable opportunities to trace developmental trajectories. While most studies focus on specific developmental time points, integrating findings from these studies may reveal the mechanisms underlying PFC cell type maturation and plasticity, providing deeper insights into its structural and functional complexity. Moreover, epigenetic modifications, which are not fully captured at the transcriptional level, are likely to play a role in PFC maturation processes. Recent studies have begun to uncover the epigenetic landscape of the postnatal PFC, including chromatin accessibility and three-dimensional chromatin interactions at the single-cell level (Lake et al., 2018; Ziffra et al., 2021; Herring et al., 2022; Yao et al., 2022; Heffel et al., 2024; Ma et al., 2024). These findings have shed light on the regulatory landscape that underpins cell type specification and maturation in the PFC. The PFC is thought to undergo a protracted maturation process, extending from birth to adolescence, relative to other cortical regions in both primates and rodents (van Eden et al., 1991; Gogtay et al., 2004; Kolb et al., 2012; Chini and Hanganu-Opatz, 2021; Kolk and Rakic, 2022). However, the mechanisms governing this protracted timeline remain poorly understood. A recent paper using human pluripotent stem cell-derived cortical neurons suggested that epigenetic regulation in neural progenitor cells sets the pace of neuronal maturation after differentiation (Ciceri et al., 2024). Future research is expected to elucidate the molecular and epigenetic factors that regulate the pace of PFC maturation and their implications for both normal development and neurodevelopmental disorders.

### Circuit formation and plasticity in prefrontal cortex development

The PFC achieves its complex brain functions through connections with various brain regions, such as the sensory cortex, ventral tegmental area (VTA), basolateral amygdala (BLA), and thalamus, with bi-directional reciprocal connections (Figure 1), being a notable feature that facilitates feedback and integration of information critical for higher-order cognitive functions (Anastasiades and Carter, 2021). While the specific roles of these circuits have been extensively reviewed elsewhere (Le Merre et al., 2021; Yizhar and Levy, 2021; Howland et al., 2022), this review focuses on the maturation of PFC neurons and the formation of circuits during postnatal development.

Neuronal maturation and circuit formation in the PFC begin during the perinatal period as cell production and migration subside (Chini and Hanganu-Opatz, 2021). During postnatal development, which progresses through the neonate, juvenile, and adolescent stages, the PFC matures more slowly than other cortical areas, such as sensory and motor areas. This prolonged maturation is observed in both primates and rodents, at least in certain aspects, including the dynamic reorganization of synaptic properties during adolescence (Paus et al., 2008; Pinto et al., 2013; Pöpplau et al., 2024) and the delayed maturation of parvalbumin-positive (PV) interneurons (Reh et al., 2020; Canetta et al., 2022). This delayed maturation of the PFC likely serves an adaptive function. Sensory and motor areas are required to develop early to process sensory inputs and produce motor outputs. In contrast, the PFC may retain plasticity during later stages to integrate sensory inputs, interpret them, and link them to motor outputs for complex behavioral decision-making. The delayed development of the PFC may also be associated with higher-order cognitive functions, such as social interactions and sexual maturation, which emerge during juvenile and adolescent periods (Chini and Hanganu-Opatz, 2021; Klune et al., 2021).

In sensory systems, it is well-established that critical periods exist during specific developmental stages when neuronal maturation and circuit formation undergo plastic changes (Hensch, 2005; Erzurumlu and Gaspar, 2012; Espinosa and Stryker, 2012; Levelt and Hübener, 2012). These critical periods allow circuits to be appropriately wired to meet environmental demands. Similarly, the PFC is thought to undergo significant plasticity during developmental stages (Larsen and Luna, 2018), which might relate to neurodevelopmental disorders, mood disorders, and addiction (Guirado et al., 2020; Nelson and Gabard-Durnam, 2020). Recent advances in optogenetic and chemogenetic tools, combined with viral vectors and genome-editing technologies, have enabled precise, circuit- or cell-type-specific manipulation of neuronal activity and gene expression at defined developmental stages. These technologies have shed light on the mechanisms underlying the progressive maturation of the PFC.

In the early postnatal stages, from the neonate to the juvenile period, distinct layer-specific changes occur in the mouse mPFC (Kroon et al., 2019). In pyramidal neurons of upper layer 3 (L3) and deep layer 5 (L5), developmental changes in dendritic morphology and intrinsic membrane properties have been observed during the first, second, and fourth postnatal weeks. While these properties develop largely in parallel across layers, excitatory inputs to L3 pyramidal neurons increase more rapidly during the second postnatal week compared to L5 neurons. Conversely, inhibitory inputs develop more rapidly in L5 than in L3. This layer-specific modulation of the excitatory/inhibitory (E/I) balance appears to be a unique feature of the PFC and is not observed in the sensory cortex. The activity of L2/3 neurons during this period drives frequency-specific spiking and enhances network oscillations within the beta–gamma frequency range, but the patterned network was not driven by the activity of L5/6 neurons in the mouse mPFC (Bitzenhofer et al., 2017). The role of neural activity during early postnatal life has also been explored in functional studies. For instance, transient optogenetic activation of excitatory neurons in L2/3 in the mPFC of neonatal mice during postnatal day 7 (P7)–P11 induces premature neuronal maturation, leading to impaired task-related gamma oscillations and deficits in PFC-dependent functions such as working memory and social preference later in life (Bitzenhofer et al., 2021) (Figure 1). These effects are thought to involve inhibitory feedback from PV interneurons. Notably, the same manipulation performed slightly later (P12–P16) results in only partial effects, suggesting the existence of a critical period for plastic changes in early postnatal development. The first one to two postnatal weeks in rodents, corresponding to the breastfeeding period, are dominated by maternal interactions. Open questions remain about the role of external stimuli, from maternal or environmental cues, in activating the PFC, whether spontaneous activity contributes, and how these factors influence long-term outcomes. Connections from the thalamus or dorsal raphe nucleus (DRN) to the PFC are already observed during this stage, with these connections potentially modulating retinoic acid (RA) and serotonin (5-HT), respectively (Larsen et al., 2019; Garcia et al., 2019). As RA plays a significant role in PFC development (Shibata et al., 2021a), external inputs in early life may regulate PFC size or compartmentalization. Following weaning, during the juvenile stage, social behaviors such as juvenile social play become prominent. In rats, this behavior, observed between P21 and P42, has been shown to influence PV-mediated inhibitory inputs and cognitive skills in the PFC later in life (Bijlsma et al., 2022).

By adolescence, sensory areas have largely matured, with significantly reduced plasticity compared to earlier developmental stages. In contrast, PFC maturation and circuit formation accelerate during this period, reflecting its extended developmental timeline and ongoing plasticity (Caballero et al., 2016, 2021). Synaptic density in the PFC undergoes dynamic changes during this period. Recent studies reveal that layer 2/3 pyramidal neuron circuits in the mouse mPFC experience transient disruption due to microglial activity during adolescence, followed by reorganization into adulthood (Pöpplau et al., 2024) (Figure 1). This reorganization is crucial for neural network formation and higher cognitive functions. High-frequency activity patterns in the PFC exhibit a biphasic trajectory during this period. The gamma and spiking activity peaks firstly during pre-juvenile (P16–P23) and early adolescence (P28–P35) but decreases significantly in late adolescence (P36-43) before rising again into adulthood (P53–P60) (Bitzenhofer et al., 2020; Pöpplau et al., 2024). Microglial phagocytic activity is increased during adolescence and promotes period-specific synaptic pruning in the PFC in mice and rats (Mallya et al., 2019; Pöpplau et al., 2024). Furthermore, microglia-driven remodeling during adolescence has been linked to not only dendritic complexity and synaptic structures but also behaviors such as social recognition memory, temporal memory, and fear of extinction later in life at the adult stage in mice (Schalbetter et al., 2022; Pöpplau et al., 2024). Adolescence thus appears to be a critical period when the PFC is particularly sensitive to microglial activity.

During adolescence, connections between the PFC and other brain regions also undergo significant establishment. Connections between the amygdala and subdivisions of the PFC, such as the ACC and IL, exhibited a marked increase during the late postweaning period in rats (Cunningham et al., 2002). Another study has shown that projections from the basolateral amygdala (BLA) and hippocampus to the PL peak at P30, then decline later at P45 into adulthood in mice. These dynamic changes are thought to influence the persistence of fear memories. Interestingly, no such peak is observed in projections to the IL, suggesting subregion-specific plasticity within the mPFC (Pattwell et al., 2016). Bidirectional connections between the mPFC and BLA also show developmental dynamics, with projections from the mPFC to the BLA peaking during adolescence and being pruned into adulthood in mice and rats (Cressman et al., 2010; Arruda-Carvalho et al., 2017). These synchronized peaks in bi-directional connectivity may optimize feedback calculations. Connections from the thalamus to the PFC, observed as early as the first postnatal week, are also highly plastic during adolescence. Chemogenetic inhibition of thalamo-prefrontal (mediodorsal/midline thalamus to medial prefrontal) activity during the postweaning period (P20–P50) impairs mPFC excitability and cognitive functions in adulthood in mice. In contrast, similar manipulations in adulthood have minimal effects, suggesting adolescence is a critical period for thalamo-prefrontal circuit development (Benoit et al., 2022). Interestingly, reduced thalamo-prefrontal connectivity has been reported in young adolescents with psychosis such as schizophrenia even before their diagnosis (Anticevic et al., 2015; Woodward and Heckers, 2016), raising the possibility that adolescence may also represent a period of heightened plasticity for modulating behaviors later in life in humans. Top-down corticocortical projections, such as those from the ACC to the visual cortex, show increased excitability during adolescence (P29–P37) in mice. Evidence suggests that disruptions to this activity during the critical developmental window may affect local and long-range input balance, leading to long-term alterations in PFC excitability and attention-related behaviors (Nabel et al., 2020; Allen and Morishita, 2024).

As described above, the PFC undergoes substantial plasticity changes during specific developmental windows for each circuit. The mechanisms driving these changes remain an active area of research. In sensory cortices, the development of inhibitory neurons is crucial for determining critical period timing (Hensch, 2005). Similarly, changes in PFC plasticity have been linked to GABAergic neurons (Caballero et al., 2016, 2021), particularly PV interneurons (Caballero et al., 2020), whose activity during adolescence (P14–P50) in the mouse mPFC has been shown to be critical for the regulation of gamma oscillations and behaviors later in life (Canetta et al., 2022). Late-adolescent activation of PV interneurons can even rescue deficits in schizophrenia model mice (Mukherjee et al., 2019). Neurotransmitters also play a crucial role in the postnatal development of the PFC, influencing its maturation and the regulation of behaviors. Key examples include serotonin, dopamine, acetylcholine, oxytocin, and endocannabinoids, each contributing to distinct aspects of PFC development (Mukherjee et al., 2019; Soiza-Reilly et al., 2019; Yaseen et al., 2019; Miguelez Fernández et al., 2021; Mastwal et al., 2023; Islam and Blaess, 2024; Molla et al., 2024; Ogelman et al., 2024) (Figure 1). For instance, serotonin signaling during the first two postnatal weeks is essential for regulating PFC maturation, including neuronal development, circuit formation, and the modulation of anxiety/depression-like behaviors in mice (Soiza-Reilly et al., 2019; Ogelman et al., 2024). In contrast, dopaminergic signaling and projections to the PFC increase significantly during adolescence, potentially correlating with pubertal changes and the refinement of cognitive functions (Walker et al., 2017b), partly through the regulation of PV interneuron maturation in mice (Mukherjee et al., 2019; Islam and Blaess, 2024). Future research should aim to clarify the precise timing, cellular targets, and behavioral outcomes of these neurotransmitter signals during critical developmental periods.

### Impact of early life stress on prefrontal cortex circuitry and function

During postnatal development, the PFC exhibits significant plasticity, facilitating the establishment of functional neural circuits. This period represents a critical window for experience-dependent brain development but also coincides with increased susceptibility to environmental factors, such as early-life stress (ELS), which may elevate the risk of mental disorders (Takeda et al., 2024; Pechtel and Pizzagalli, 2011; McLaughlin et al., 2010; Green et al., 2010; Nestler and Russo, 2024). Evidence from human studies indicates that childhood neglect or abuse is associated with deficits in social–emotional development and cognitive functions, including IQ, memory processing, and problem-solving abilities (Egeland et al., 1983; Nelson et al., 2007; Spatz Widom et al., 2007; De Bellis et al., 2009; Pollak et al., 2010). Alterations in synaptic dynamics during critical developmental periods have been implicated in the pathophysiology of neurodevelopmental disorders, such as autism spectrum disorder and schizophrenia (Forrest et al., 2018; Faust et al., 2021). Recent studies using rodent models have provided valuable insights into the mechanisms that render the developing brain particularly vulnerable to early-life stress (ELS) and its contributions to the onset of mental disorders later in life. This review focuses primarily on the impact of social stressors, including maternal neglect and social isolation, on the structural and circuit-level development of the PFC.

**Figure 2:**
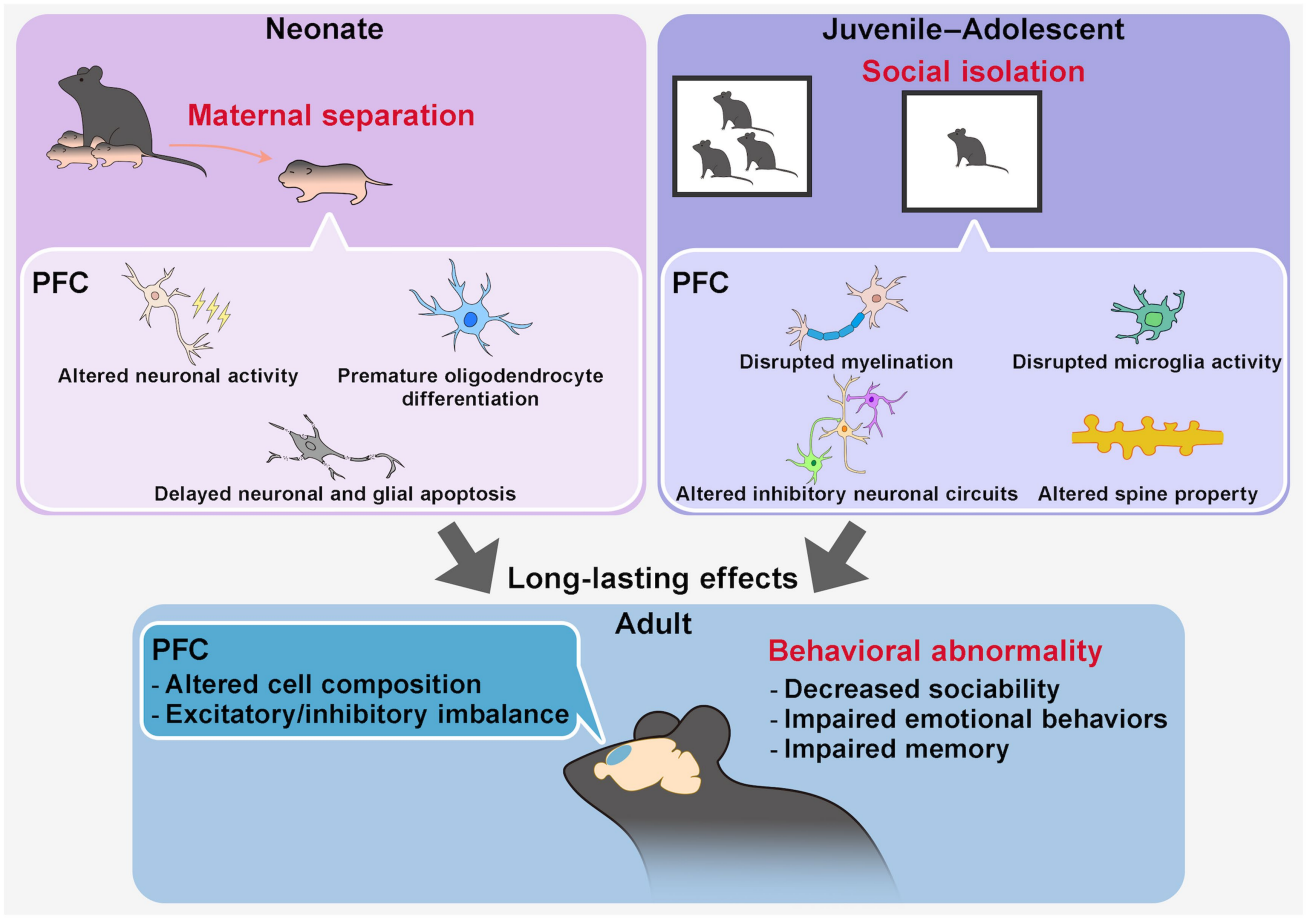
Long-lasting effects of early-life social stress on PFC structure and function. Early-life social stress, such as maternal separation during the neonatal period or social isolation during juvenile–adolescent stages, has been widely used as a model for early-life stress (ELS). These stressors induce persistent alterations in PFC structure, circuit properties, and behaviors into adulthood. During the stress period, maternal separation in neonates affects PFC neuronal activity and cell composition, whereas social isolation in juveniles and adolescents influences neuronal activity, myelination, and microglial function. These changes have been implicated in long-term disruptions in PFC circuit organization, including cell composition and excitatory/inhibitory (E/I) balance, in a circuit- and PFC subregion-specific manner. Ultimately, ELS contributes to behavioral abnormalities in adulthood, including impairments in sociability, emotion regulation, and memory function.

ELS in neonatal rodents is frequently modeled using maternal separation (MS) or environmental deprivation (e.g., limited bedding and nesting materials), both of which disrupt caregiving environments and lead to behavioral alterations in adulthood, including impaired learning and memory, social deficits, and increased depression-like behaviors (Tractenberg et al., 2016; Walker et al., 2017a). MS has been shown to influence cytoarchitecture by impairing oligodendrocyte differentiation in the mouse mPFC (Teissier et al., 2020) and delaying the onset of neuronal and glial apoptosis in the rat mPFC (Majcher-Maślanka et al., 2019), ultimately altering the composition of each cell type in adulthood. Functionally, it has been reported that MS reduces neuronal activity in the PFC during the stress period. Chemogenetic reduction of mPFC excitability during the first two postnatal weeks (P2–P14) in mice reared under standard facility conditions replicates MS-associated phenotypes, including premature oligodendrocyte differentiation and impairments in emotional behavior and object recognition in adulthood, underscoring a critical period for stress susceptibility (Teissier et al., 2020) (Figure 2). The presence of this critical period is further supported by a study showing that transient chemogenetic activation of PFC neurons during MS in mice ameliorates behavioral deficits in adulthood (Menezes et al., 2024). However, MS has also been reported to increase neuronal excitability, reduce inhibitory neurons, and shift the excitatory/inhibitory (E/I) balance toward excitation in the PFC in mice and rats (Ohta et al., 2020; Oh et al., 2021). Whether stress increases or decreases excitability may depend on specific neuronal subtypes, cortical layers, and circuit connections. Moreover, differences in stress sensitivity have also been reported in circuits between the PFC and specific brain regions (Reincke and Hanganu-Opatz, 2017; Oh et al., 2021) as well as across subregions within the PFC (Luo et al., 2023).

During juvenile and adolescent periods, social isolation is a commonly used stress model to examine long-term effects on PFC function in rodents (Burke et al., 2017; Li et al., 2021; Morishita, 2021). Juvenile social isolation (jSI), particularly during the postweaning period between P21 and P35 in mice, induces greater deficits in social behaviors and cognition in adulthood compared to later isolation periods (P35-P65), indicating a critical period of heightened stress sensitivity during early adolescence (Makinodan et al., 2012). jSI disrupts key elements of neuronal plasticity, including myelination and microglial activity (Makinodan et al., 2012, 2017; Komori et al., 2024), and alters the organization of circuits involving PV or somatostatin (SST)-positive interneurons, leading to long-term changes in E/I balance and behavior (Bicks et al., 2020; Yamamuro et al., 2020; Li et al., 2022) (Figure 2). Social isolation during adolescence in mice also influences actin dynamics in the PFC, which is also observed in rat MS model (Tada et al., 2016), activating cytoskeletal regulatory pathways typically suppressed during this period, thereby affecting dendritic spine properties and long-term brain function (Li et al., 2024a). Furthermore, PFC-associated circuits, including PFC-posterior paraventricular thalamus (pPVT), PFC-nucleus accumbens (NAc), PFC-VTA, and PFC-BLA pathways, have been implicated in mediating the long-term effects of isolation stress in early life on social and cognitive behaviors in adulthood in rodents, with neurotransmitters like dopamine and subregion-dependent differences playing important roles (Baarendse et al., 2013; Yamamuro et al., 2020; Park et al., 2021; Kuniishi et al., 2022; Li et al., 2022; Musardo et al., 2022; Wang et al., 2022).

### Epigenetic changes in the prefrontal cortex induced by early life stress: emerging insight

Comprehensive transcriptomic analyses have increasingly been employed in recent years to investigate how ELS influences genome-wide gene expression in the PFC of rodents (Peña et al., 2019; Usui et al., 2021; Wang et al., 2022; Luo et al., 2023). For example, single-cell transcriptomic analysis of PFC tissues from adult mice exposed to MS has revealed long-lasting alterations in gene expression, particularly in genes related to GABAergic and serotonergic pathways (Ohta et al., 2020; Menezes et al., 2024). Environmental factors are thought to influence epigenetic states, which may underlie these persistent transcriptional alterations. Recent studies have shown that stress during early life or adulthood alters histone modifications and DNA methylation states at loci such as *Bdnf* or serotonin-related genes in the rodent PFC, which are crucial for neuronal plasticity (Roth et al., 2009; Márquez et al., 2013; Xu et al., 2018; Konar et al., 2019; Zhao et al., 2020; Fachim et al., 2021; Jiang et al., 2021; Araki et al., 2024). Moreover, ELS has been shown to induce long-lasting epigenetic changes in cell types critical for neural plasticity, such as PV interneurons and oligodendrocytes, within the rodent PFC (Chen et al., 2020; Noel et al., 2024). Pharmacological inhibition of histone-modifying enzymes, including histone H3K9 methyltransferase, histone H3K4 demethylases, and histone deacetylases (HDACs), has been shown to mitigate behavioral abnormalities caused by ELS in rodent models, suggesting that epigenetic regulation may play a role in both the pathogenesis and potential treatment of stress-related disorders (Wei et al., 2020; Wang et al., 2023; Hernandez Carballo et al., 2024; Li and Yan, 2024). Notably, these inhibitors were administered not only during the stress exposure itself but after the stress period or even later in adulthood, yet still resulted in significant behavioral improvements. This highlights the enduring nature of stress-induced epigenetic changes and their potential reversibility. Furthermore, recent studies in mice suggest that susceptibility to social stress may also be linked to the regulation of gene expression by specific histone-modifying enzymes in the PFC and NAc (Kronman et al., 2021; Li et al., 2024b; Torres-Berrío et al., 2024). Future research should aim to elucidate the specific timing, cell types, and epigenetic mechanisms underlying stress responses to better understand how ELS shapes long-term PFC function and stress vulnerability.

## Discussion

This review has highlighted studies, primarily in rodents, illustrating how the PFC undergoes sequential structural and circuit development during postnatal stages. Particular attention has been given to periods of elevated plasticity in the PFC, during which circuit formation and reorganization are especially dynamic. Furthermore, we discussed how these critical windows of plasticity render the PFC particularly vulnerable to environmental stressors, such as ELS, and explored the relationship between ELS and epigenetic regulation in shaping PFC function.

Emerging evidence indicates that the effects of ELS differ substantially across developmental stages, cell types, PFC subregions, and circuits, highlighting the necessity for more detailed investigations. Notably, while the impact of ELS persists into adulthood, epigenetic modifications induced by stress appear to continue evolving even in mature stages. Future research should focus on identifying the genes susceptible to long-term epigenetic changes and elucidating how these alterations influence PFC function over time.

Interestingly, the long-lasting effects of ELS are also implicated in the vulnerability to subsequent stress exposure, consistent with the “two-hit” model (Castillo-Gómez et al., 2017). Differences in responses to second-hit stress have been linked to epigenetic mechanisms, which can either increase or decrease stress resilience depending on the context (Chaby et al., 2020; Reshetnikov et al., 2021). Additionally, even in fully developed adult mice, the PFC retains experience-dependent plasticity (Levy et al., 2019), suggesting that future studies should not only address second-hit vulnerabilities but also investigate the factors that determine plasticity in the adult PFC.

This review did not extensively address how findings from rodent studies translate to humans. While the structural and circuit-level correspondence between the rodent and primate PFC has been discussed extensively (Carlén, 2017; Chini and Hanganu-Opatz, 2021; Le Merre et al., 2021; Preuss and Wise, 2022), further investigation is needed to clarify the similarities in stress responsiveness, particularly given the differences in developmental timelines. Despite the significant differences in the extent of postnatal development, it is worth noting that PFC development appears to progress more slowly than other cortical areas in both humans and rodents, raising intriguing questions about the mechanisms that contribute to the protracted maturation of the PFC.

Another limitation of this review is the lack of discussion on sex differences. Notably, sexual maturation occurs during the juvenile and adolescent periods, and numerous studies have emphasized the sex-specific effects of stress on PFC development and function (Tottenham and Galván, 2016; Heitzeg et al., 2018; Schroeder et al., 2018; Konar et al., 2019; Shaw et al., 2020; Wang et al., 2022). Future research should seek to unravel the intricate interplay between sex differences, developmental stages, and stress responses in shaping PFC development and function.

Achieving a comprehensive understanding of PFC development and its vulnerability to stress will require integrating data across multiple dimensions. By bridging single-cell transcriptional and epigenetic states with circuit-level changes and behavioral outcomes at distinct developmental stages, future research holds the potential to uncover the mechanisms that drive PFC maturation and contribute to the pathophysiology of psychiatric disorders.
